# Design and development of the Australian and New Zealand (ANZ) myeloma and related diseases registry

**DOI:** 10.1186/s12874-016-0250-z

**Published:** 2016-11-09

**Authors:** Krystal Bergin, Elizabeth Moore, Zoe McQuilten, Erica Wood, Bradley Augustson, Hilary Blacklock, Joy Ho, Noemi Horvath, Tracy King, John McNeil, Peter Mollee, Hang Quach, Christopher M. Reid, Brian Rosengarten, Patricia Walker, Andrew Spencer

**Affiliations:** 1 Department of Epidemiology and Preventive Medicine, Monash University, Melbourne, VIC Australia; 2 The Alfred Hospital, Melbourne, VIC Australia; 3 Sir Charles Gairdner Hospital, Perth, WA Australia; 4 Middlemore Hospital, Middlemore, New Zealand; 5 Royal Prince Alfred Hospital, Sydney, NSW Australia; 6 Royal Adelaide Hospital, Adelaide, SA Australia; 7 Princess Alexandra Hospital, Brisbane, QLD Australia; 8 St Vincent’s Hospital Melbourne, Melbourne, VIC Australia; 9 Myeloma Foundation Australia, Melbourne, VIC Australia; 10 Peninsula Health, Frankston, VIC Australia; 11 School of Public Health, Curtin University, Perth, WA Australia; 12 South Block, Alfred Health, 55 Commercial Road, Melbourne, VIC 3004 Australia

**Keywords:** Plasma cell dyscrasia, Multiple Myeloma, Registry, Real-world, Development, Implementation

## Abstract

**Background:**

Plasma cell dyscrasias (PCD) are a spectrum of disorders resulting from the clonal expansion of plasma cells, ranging from the pre-malignant condition monoclonal gammopathy of undetermined significance (MGUS) to multiple myeloma (MM). MM generates a significant burden of disease on the community and it is predicted that it will increase in both incidence and prevalence owing to an ageing population and longer survival secondary to new therapeutic options. Robust and comprehensive clinical data are currently lacking but are required to define current diagnostic, investigational and management patterns in Australia and New Zealand (ANZ) for comparison to both local and international guidelines for standards of care. A clinical registry can provide this information and subsequently support development of strategies to address any differences, including providing a platform for clinical trials. The Myeloma and Related Diseases Registry (MRDR) was developed to monitor and explore variations in practices, processes and outcomes in ANZ and provide benchmark outcomes nationally and internationally for PCD. This paper describes the MRDR aims, development and implementation and discusses challenges encountered in the process.

**Methods:**

The MRDR was established in 2012 as an online database for a multi-centre collaboration across ANZ, collecting prospective data on patients with a diagnosis of MGUS, MM, solitary plasmacytoma or plasma cell leukaemia. Development of the MRDR required multi-disciplinary team participation, IT and biostatistical support as well as financial resources.

**Results:**

More than 1250 patients have been enrolled at 23 sites to date. Here we describe how database development, data entry and securing ethics approval have been major challenges for participating sites and the coordinating centre, and our approaches to resolving them. Now established, the MRDR will provide clinically relevant and credible monitoring, therapy and ‘real world’ outcome data, to support the conduction of high quality studies. In addition, the Myeloma 1000 sub-study is establishing a repository of paired peripheral blood specimens from registry patients to study mechanisms underlying disease progression.

**Conclusion:**

Establishment of the MRDR has been challenging, but it is a valuable investment that will provide a platform for coordinated national and international collaboration for clinical research in PCD in ANZ.

## Background

Plasma cell dyscrasias (PCD) are a spectrum of disorders resulting from the clonal expansion of terminally differentiated B-cells known as plasma cells. PCD range from the pre-malignant monoclonal gammopathy of undetermined significance (MGUS) to multiple myeloma (MM). Patients with MGUS have a risk of progression to MM of 1 % per year [1]. MM is the second most common haematological malignancy, has a significant community disease burden [2, 3] and is predominantly a disease of the elderly with a median age at diagnosis of approximately 70 years [4]. While the overall prognosis for MM remains poor and the disease is considered incurable with pharmacotherapy, the introduction of new therapies including proteasome inhibitors (PI) and immunomodulatory drugs (IMID) has resulted in a significant improvement in MM outcomes, including the duration of survival [5–8]. Improved survival combined with an ageing population is predicted to result in an increase in both the incidence and prevalence of MM in the next few decades.

Currently, limited robust and comprehensive clinical data exist, including demographics, current management and outcomes for patients with MM and related diseases in Australia and New Zealand (ANZ). These data are required to define the current diagnostic, investigational and treatment patterns in ANZ for comparison to both local and international guidelines for standards of care. A clinical registry can provide this information and subsequently support development of research and strategies to address any differences and assess their impact on clinical outcomes.

### Clinical registries

Population-based cancer registries are well established in Australia [9, 10]. These registries provide broad population health surveillance and research support, however, they typically do not provide sufficient detail for accurate staging, treatment or outcome data with the exception of survival. Cancer Australia has labelled these data ‘fundamental’[9] and in November 2010, in answer to this unmet need, the Australian Health Ministers’ Conference (AHMC) endorsed the “Strategic and Operating Principles for Australian Clinical Quality Registries”, for a national approach to Australian clinical quality registries [11, 12]. A clinical-quality registry can be defined as a dataset of pre-defined information from a group of patients with a particular disease, using a particular health care resource or undergoing a particular procedure [13, 14]. Clinical registries are able to provide high quality data allowing monitoring of health care utilisation and therapy patterns as well as detailed outcome data. These data are useful in benchmarking clinical outcomes over large geographical areas particularly in rare conditions where data from other sources are often lacking.

All new histological diagnoses of cancer in ANZ are required to be reported to state-based cancer registries along with mortality data. These data, although valuable, are inadequate for the analysis of treatment patterns or therapy response. Currently there is no National Minimum Data Set for cancer-related data; however, ‘The Cancer (clinical) Data Set Specification’ is recommended as best practice [15]. Linkage of these data to more extensive clinical and psychosocial data allows enhancement of service provision, treatments and outcomes for patients affected by these conditions [16]. This is particularly important in the case of PCD as the diagnosis requires consideration of both clinical and pathological features. As such, the differentiation between the various PCD is not possible based on the data available to the state-based cancer registries, limiting the validity of any survival analyses and other assessments [17].

The Myeloma and Related Diseases Registry (MRDR) was developed to monitor and explore variations in practices, processes and outcomes in the ANZ setting and provide benchmark outcomes nationally and internationally for PCD. The registry is also intended to act as a resource for future clinical trials and projects such as M1000, a MRDR sub-study which is establishing a repository of paired peripheral blood specimens from registry patients to study mechanisms underlying disease progression. This article describes the aims, development and implementation of the MRDR and discusses some of the challenges experienced in the process.

## Methods

### Implementation

Clinical registries, although immensely useful, are difficult to initially develop and implement, requiring the involvement of a multi-disciplinary team, including at a minimum, patient groups, data collectors, clinicians, information technology (IT) and allied health staff. Biostatistical support is also mandatory to enable the robust analysis of acquired data. Ideally a clinical registry would collect all data for the population of interest at all time points, however, the comprehensiveness of a database needs to be weighed against the simplicity of use for the overall best outcome. A suggested timeline for the development of a clinical registry is shown in Table 1.

**Table 1:** Suggested timeline for development of a clinical registry

Pre-development Phase (0–6 months)
• Secure funding
• Finalise project plan
• Establish Steering Committee
Development phase (6–12 months)
• Finalise data set and data dictionary
• Web-database construction
• Establish contacts at 4–6 pilot hospitals
• Ethics submission at selected academic unit or central site and pilot sites
Implementation Phase (1 year onwards)
• Data collection commences at 4–6 pilot sites
• Identify additional sites for inclusion in the Registry
Expansion Phase (18 months onwards)
• Ethics submission at additional sites
• Data collection commences at additional sites
• Test and develop new or improved measures of outcome

### Resources

A team of researchers and collaborators with clinical, research and epidemiological expertise was assembled to develop the study concepts and to contribute to initial project scoping, including data items to be collected, pilot sites to be approached, and plans for analysis of registry data. A partnership with Monash University was established through the Department of Epidemiology and Preventive Medicine (DEPM), which has an internationally recognised academic epidemiology unit with expertise in registry development, informatics and management of similar clinical registries in ANZ.

Not infrequently clinical registries are established prior to the confirmation of the resources required, relying on the good will of clinical and clerical staff for data entry, in addition to their existing workload. Personnel time is not an insignificant resource requirement for the MRDR as data entry requires approximately 45–60 min for a new patient and 15–30 min for each review thereafter. Payment to hospital sites for complete data on registered patients is ideal but may not be feasible.

At inception the major initial requirements for the MRDR were administrative and financial. Procurement of funding for clinical registries is difficult as they do not fit into traditional research funding schemes designed for clinical trials, therefore, investigators need to source funding via new and innovative methods. Funding required for clinical registries is unique to the individual needs of a particular registry. In general, costs to consider when planning a registry budget include staff time for project management, submissions for human research ethics committee approvals, initial database development and maintenance, data entry and management, case ascertainment and data linkage costs, and preparation of reports to participating sites. Seed funding for the MRDR was secured via grants from both the pharmaceutical industry and Myeloma Australia leveraging established links with the clinical experts involved in the creation of the registry. These funds were used to design and build an individualised, web-based software platform with a user-friendly interface and were sufficient for the initial set up and the first 2–3 years of registry operations. It was recognised that a commitment of ongoing financial support from government agencies would be desirable and that this would require the demonstration of quality improvement in the diagnosis and management of PCD for patients and the health care sector. This will be achieved through reports and publications of registry data with the potential to translate into both policy and practice change.

### Database architecture and design

The MRDR is a web based application and the security and robustness of the system is determined by its architecture, servicing and monitoring. Data is stored on a separate secure server to the web application providing additional data security should the web server be breached. There is restricted access with a unique access port to the web server controlled by firewalls. An IP Sec tunnel between servers ensures a secure conduit for all data which are encrypted with strong secure sockets layer (SSL) encryption. The system requires continual servicing and monitoring with service patches to ensure there are no vulnerabilities in the architecture. Periodic penetration testing and regular updates of the authorised user access list are undertaken with security logging of attempts to access the system and audit logs of database changes to help to identify suspicious activity.

### Governance

A steering committee was established to define clear objectives and provide project and research guidance and oversight. Members were selected to provide guidance on all aspects integral to the development, implementation and management of the registry. Members include haematologists with track records of leadership and expertise in the management of MM, from the different regions likely to be involved in the registry, a specialist MM nurse consultant, a MM consumer group representative and researchers and a database expert from the DEPM. In conjunction with an operations committee, the steering committee facilitated the design, content and structure of the registry utilising the varied skill sets of individual members. The steering committee meets every 3–4 months via teleconference to discuss progress and plans for continued registry development in addition to any issues reported by the sites. The full terms of reference of the steering committee are shown in Table 2.

**Table 2:** Terms of reference of the Steering Committee

Terms of reference of the Steering Committee include:
• Monitor the scientific progress of the project including the data quality
• Advise on the collection and interpretation of data
• Assess and advise regarding performance outliers
• Advise on scientific priorities to be addressed in data analysis and publication strategy
• Review publications of the project and advise on their scientific quality
• Review all research and external data requests

### Inclusion and exclusion criteria

Inclusion and exclusion criteria were selected by the steering committee to be as inclusive of target MM and related disease cases as possible while still specific enough to produce usable and robust data. Cases were initially defined as incident cases of “myeloma or a related disease” in accordance with the International Myeloma Working Group (IMWG) diagnostic criteria [17]. Minimal revisions of the definition of “myeloma or a related disease” by the steering committee members with clinical expertise were required to improve ease of data collection with the only major change being the removal of systemic amyloidosis and specific inclusion of plasma cell leukaemia (PCL). “Myeloma or a related disease” was ultimately defined as one of MGUS, MM, solitary plasmacytoma, or PCL. An incident case was defined as a diagnosis of MM or a related disease or a death attributable to MM or a related disease with diagnosis no more than 3 months prior to ethics approval at the respective site to ensure the integrity of the registry as a prospective database. While ensuring the capture of a meaningful proportion of MGUS patients at any particular site is difficult, as data from this patient population are limited through other avenues, it is of particular interest, especially in relation to progression to symptomatic MM. Other non-disease specific inclusion and exclusion criteria were minimised to facilitate a cohort that is as complete and representative of real life practice as possible. These criteria only stipulated an adult population (age ≥18 years) who had not chosen to ‘opt off’ the registry (see “Ethics” below). Final acceptance of the inclusion and exclusion criteria was approved by the steering committee led by the principle investigator.

### Data selection

Data item selection for inclusion on a clinical registry is a critical component that defines the quality and clinical usefulness of the eventual output. The clinical research expertise of the steering committee with track records in investigator-initiated clinical trials and database development and use, informed the content of a proposed practical and useful dataset. Usefulness of the dataset was primarily judged by predicted ability of outcome data extraction by the clinicians on the steering committee including demographic variables, treatment details and clinical, radiological and laboratory variables required for therapy response assignment. These variables were then discussed with all members of the steering committee to ensure the final dataset would be both clinically useful and practical for data collectors as well as suitable for use with the IT software to produce useful data extraction results. In addition, discussion with other research groups involved in established clinical registries provided insights into clinical data of importance for inclusion to avoid preventable future missing data. Great care was taken in configuration of the interface of the database to ensure user-friendliness with drop-down boxes favoured for categorical data, and minimal areas requiring free text. Decisions on the therapy data required were guided by both clinical utility and availability for collection by non-medically trained data-entry staff. Data selected for inclusion included demographic data, diagnosis data, therapy and supportive care administered, disease responses and survival (shown in Table 3).

**Table 3:** Key Data Entry Time Points and Data Items

Key Data Entry Time Points	Data items collected:
Baseline demographics at diagnosis	• Health at diagnosis • Demographic Details • Laboratory and imaging results at diagnosis
Changes in treatment regimen	• Therapy decisions including including pre therapy benchmarking, chemotherapy, autologous stem cell transplants, allogeneic stem cell transplants and maintenance and supportive therapy
Treatment regimen dates	• Commencement and completion for each agent/therapy
Progression or disease relapse	• Myeloma markers as per IMWG criteria • Date of disease progression or relapse
Treatment response	• Response as per IMWG criteria • Outcomes (overall and progression free survival, duration of response and time to next treatment and quality of life measures – EQ-5D-5L)
Date (and cause) of death	• Long-term outcomes (through linkage with Cancer and Death Registries).

MM and related diseases represent a complex niche area of haematology that many data collectors may not be familiar with, resulting in the possibility of inaccurate entry, particularly with respect to therapeutic responses. To counter this, extra data points were collected to enable independent assessment of response by MRDR MM experts. This information was then converted into a functional online database by a professional clinical informatics team that is accessed directly by participating sites.

Clinical registries such as the MRDR are by nature non-interventional and dynamic, describing the natural history of the disorders and therapies used in their management over time. MM typically relapses following treatment and remains incurable with current therapies. Key time points based on a typical patient’s diagnostic process, subsequent initiation of treatment and routine follow-up were agreed upon by a consensus of opinion of the clinical members of the steering committee. Registry outputs were to include detailed patient and disease demographics, treatment selection and delivery, and subsequent correlation with disease response (see Table 3). Due to the clinically heterogeneous and relapsing nature of MM these time points may be difficult to predict implying that continual data collector review is required. To overcome this, a review period of 4 months for MM and PCL and annually for MGUS and solitary plasmacytoma, reflecting the natural history of these disorders, was agreed upon to balance the risk of missing important information while ensuring a manageable workload for hospital data collectors. The choice of these review periods has ensured data collection is not excessively onerous, necessitating data collectors on only a part time basis for most sites. As the MRDR matures with larger numbers of patients registered requiring ongoing review these personnel resource needs will increase and review periods may need to be reassessed and modified.

It is hoped that with time larger numbers of patients will be registered on the MRDR with maturing data creating significant research output. These outputs will ideally result in funding opportunities providing sites with further financial support for ongoing data collection for both new patient registrations and follow up for existing patients.

## Results

### Current status

The Australia and New Zealand Myeloma and Related Diseases Registry was established in 2012. Currently there are 23 approved sites, including 20 Australian and three New Zealand sites, with nine more sites in the process of obtaining ethical approval to participate as of September, 2016. Patient registrations reached 1000 in March, 2016 with over 1250 patients enrolled as of September, 2016.

### Research output and communication

The MRDR uses several forums to update stakeholders, share information with supporters and collaborators, and invite participation. Regular updates are included in publications for myeloma patient and specialist clinician groups and the registry circulates a periodic newsletter for interested parties including clinicians, nursing staff, patients, industry and community sponsors. An annual MRDR interest group breakfast meeting is held at the major ANZ haematology conference and MRDR data has also been presented at national and international medical conferences.

Analysis of registry data is undertaken by MRDR staff working in the DEPM at Monash University. These data are interpreted with the input of specialist clinicians on the steering committee. A PhD student associated with the MRDR contributes to clinical input, analysis and publication. The first comprehensive evaluation of the epidemiology and current treatment of MM in ANZ using registry data is in preparation for publication.

## Discussion

### Case identification and registration

The aim of the MRDR is the creation of a population-based registry to monitor and explore variation in practices, processes and outcomes for PCD in ANZ, and to provide data for benchmarking nationally and internationally. Initial patient registration is completed by clinicians after identifying appropriate patients in the clinic. A truly population-based registry requires as close as possible to 100 % coverage of all sites that manage PCD in ANZ, both in the public and private sector. Sites where members of the steering committee held clinical appointments were the first sites to register patients, then further sites suggested by steering committee members were approached. Once the profile of the registry grew clinicians began to actively seek involvement. However, to improve the inclusiveness and clinical usefulness of data, under-represented areas including non-Victorian, rural and private sector sites are now prioritised. Participation by these sites is being actively sought by direct email contact and networking at local, national and international meetings and conferences. Additional assistance for site set-up is also provided by the MRDR team including a mock online database for demonstration of the required data input, and support with ethics submissions.

### Quality control - case ascertainment and audits

Case ascertainment at participating sites will be monitored through linkage with state and national cancer registry data, with missing or discrepant cases followed up with local sites to ensure that close to 100 % of cases are registered. Comparison of patients registered on the MRDR with patients registered on the Victorian Cancer Registry from participating Victorian sites is underway. As the number of interstate sites grows, arrangements with other state based cancer registries are being pursued. In addition, sites’ ability to access their own data for review facilitates data completion and the opportunity for local audits. Completeness of key data points is assessed and fed back to sites in biannual reports, promoting and enhancing data completeness and quality. Audits of 5 % of registry cases against source data will also assess accuracy and completeness of data collection.

### Clinical feedback for participating sites

Benefits of involvement with the MRDR include clinical feedback on data entered. Initially data was only provided to sites in aggregate form. Feedback from the sites to the steering committee suggested that this did not allow individual sites sufficient information to reflect on their own management practice. This prompted a change, with an online data extraction link provided to all sites, which allows individual sites access to their own data. This allows them to evaluate their practice and provides an incentive for data completion and ongoing registry participation.

### Continuity of patient access

An advantage of patient registration on the MRDR lies in its ability to provide comprehensive clinically useful data on cohorts of patients. In real life clinical practice if a patient is referred to another centre for ongoing care the patient is often lost to follow up by the initial treating clinician. The recognition of this has enabled a recent modification to the database that now permits transfer (and return) of patient data from one centre to another within the registry. This allows clinicians to assess the treatment and outcome data of their patients even after referral and ensures that the patient will be included in the corresponding site’s biannual reports, thus enabling each site to maintain an accurate and complete picture of their patient cohort.

### Ethical and privacy considerations

Involvement of multiple sites requires ethics approvals from the Human Research Ethics Committee (or equivalent) at each institution. Privacy legislation in Australia allows ‘opt off’ consent models for activities like registries where the minimal impingement on individual privacy rights from participation is likely to be outweighed by the public interest of having data available [18]. For simplicity and maximum participation such an ‘opt off’ consent model was adopted, with clinicians inviting patient participation in the registry and providing a written brochure. The information brochure describes the nature and purpose of the registry and requirements for involvement. Contact details and information to ‘opt off’ the registry are also provided. Only two patients have chosen to ‘opt off’ at the time of writing. This may reflect the fact that the data collected does not exceed that routinely collected by clinicians for direct patient management, therefore participation is not perceived by patients as being onerous or intrusive.

Ethics submissions in ANZ can be lengthy and time-consuming processes, creating further strain on the already stretched financial and personnel resources of individual sites. Some hospital ethics committees are unfamiliar with the ‘opt off’ model, as it is very different from the framework used for clinical trials, and some have been hesitant to approve such submissions. However, the MRDR steering committee and Project Manager provide practical, hands-on support to sites in the preparation of materials for, and management of ethics submissions. Streamlined ethics processes already in place for clinical trials in ANZ have recently been extended to registries, which will help future site recruitment and initiation.

Requirements for stakeholders wishing to store identified data on New Zealanders in overseas hosted services have changed recently. An accepted overseas-based cloud or hosting service must be used, or alternatively an exemption must be obtained.

### Further research

Sites have received their first reports providing a summary of key clinical performance and quality indicators for their sites in comparison with overall results for all patients on the MRDR. This information will facilitate local comparative evaluation to support practice improvement and over time, change in practice at sites will be monitored and evaluated to assess the impact of site reports. In addition, sites can analyse, present and publish their own data from the MRDR. Other parties may apply for data for research purposes, in accordance with MRDR Data Access Policy guidelines.

One of the key aims of the registry is to act as a resource for further research. Proposals for MRDR-related studies are invited regularly by email and inclusion in the regular newsletter from all parties involved with or with an interest in the MRDR. Several initial propositions made in response to these invitations are currently in development. International collaborations with sister PCD registries in other countries have been fostered during networking opportunities created by attendance at key international meetings. Links were initially established at the International Myeloma Workshop in Rome in 2015, and then further consolidated with a proposal currently under development for a comparative study on health-care models, first-line therapy and response in MM in collaborating countries. International data sharing and security issues may prove to be an obstacle to participation for some countries.

### Myeloma 1000

The Myeloma 1000 project, a sub-study of the MRDR, aims to establish a repository of 1000 fully annotated peripheral blood specimens from MGUS patients and a further 1000 from MM patients. Samples are collected from consenting participants on the registry at the time of diagnosis, after completing a separate consent with an ‘opt in’ model by qualified staff not directly associated with the registry. Newly diagnosed patients appropriate for this sub-study are identified by treating clinicians at each site prior to initiation of any treatment. After appropriate initial discussion with the treating clinician, formal consent for participation is obtained. Peripheral blood samples are collected on site using pre-prepared kits and returned to the MRDR using the express post packs provided for inclusion in the biobank at no cost to the sites. These samples will be available for future correlative studies including, the study of circulating biomarkers and molecular epidemiological questions. Through linking prospective, long-term clinical data with biological data provided by these stored samples it is anticipated that the Myeloma 1000 project will provide insight into the mechanisms underlying disease progression so as to inform optimal treatment strategies for MM and related diseases. Currently the Myeloma 1000 project has stored samples from 46 MM and 55 MGUS patients accrued over a period of 20 months.

## Conclusions

The benefits of clinical registries are well established. By providing a clinically relevant and credible means of monitoring patient populations, therapies and outcomes, they enable high quality studies of real world data in even rare diseases. Prospectively collected datasets that are manageable for data collectors without compromising the complexity and comprehensiveness of data provide ample opportunities for integration and analysis to address a multitude of clinical questions currently and into the future. Establishing a national registry is challenging, but the MRDR is a valuable ANZ resource with data on over 1250 patients to date and with potential for national and international research collaborations.

## Abbreviations

AHMC: Australian health ministers’ conference
ANZ: Australia and New Zealand
DEPM: Department of epidemiology and preventive medicine
IMID: Immunomodulatory drugs
IMWG: International myeloma working group
IT: Information technology
MGUS: Monocolonal gammopathy of undetermined significance
MM: Multiple myeloma
MRDR: Myeloma and related diseases registry
PCD: Plasma cell dyscrasia
PCL: Plasma cell leukaemia
PI: Proteasome inhibitors
SSL: Secure socket layer
